# Sub-millimeter variation in human locus coeruleus is associated with dimensional measures of psychopathology: An *in vivo* ultra-high field 7-Tesla MRI study

**DOI:** 10.1016/j.nicl.2019.102148

**Published:** 2020-01-15

**Authors:** Laurel S. Morris, Aaron Tan, Derek A. Smith, Mora Grehl, Kuang Han-Huang, Thomas P. Naidich, Dennis S. Charney, Priti Balchandani, James W. Murrough, Prantik Kundu

**Affiliations:** a Depression and Anxiety Center for Discovery and Treatment, Department of Psychiatry, Icahn School of Medicine of Mount Sinai; b BioMedical Engineering and Imaging Institute, Icahn School of Medicine of Mount Sinai; c Department of Neurosurgery, Icahn School of Medicine of Mount Sinai; d Department of Pediatrics, Icahn School of Medicine of Mount Sinai; e Office of the Dean, Icahn School of Medicine of Mount Sinai

**Keywords:** Anxiety, PTSD, High-field MRI, Locus coeruleus, Structural imaging, Norepinephrine

## Abstract

The locus coeruleus (LC) has a long-established role in the attentional and arousal response to threat, and in the emergence of pathological anxiety in pre-clinical models. However, human evidence of links between LC function and pathological anxiety has been restricted by limitations in discerning LC with current neuroimaging techniques. We combined ultra-high field 7-Tesla and 0.4 × 0.4 × 0.5 mm quantitative MR imaging with a computational LC localization and segmentation algorithm to delineate the LC in 29 human subjects including subjects with and without an anxiety or stress-related disorder. Our automated, data-driven LC segmentation algorithm provided LC delineations that corresponded well with postmortem anatomic definitions of the LC. There was variation of LC size in healthy subjects (125.7 +/- 59.3 mm^3^), which recapitulates histological reports. Patients with an anxiety or stress-related disorder had larger LC compared to controls (Cohen's *d* = 1.08, *p* = 0.024). Larger LC was additionally associated with poorer attentional and inhibitory control and higher anxious arousal (FDR-corrected p's<0.025), trans-diagnostically across the full sample. This study combined high-resolution and quantitative MR with a mixture of supervised and unsupervised computational techniques to provide robust, sub-millimeter measurements of the LC *in vivo,* which were additionally related to common psychopathology. This work has wide-reaching applications for a range of neurological and psychiatric disorders characterized by expected LC dysfunction.

## Highlights

• We combined ultra-high field 7-Tesla and 0.4 × 0.4 × 0.5 mm quantitative MR imaging with a computational LC localization and segmentation algorithm.
• LC was delineated in 29 human subjects including subjects with and without an anxiety or stress-related disorder.
• Patients with an anxiety or stress-related disorder had larger LC compared to controls (Cohen's *d* = 1.08, p = 0.024).
• Larger LC was additionally associated with poorer attentional and inhibitory control and higher anxious arousal (FDR-corrected p's<0.025), trans-diagnostically across the full sample.

## 1 Introduction

Pathological anxiety can be defined as an excessive, maladaptive fear-like state, which acts as a major risk factor for suicide (Rosen and Schulkin, 1998; Eysenck, 1992; Kessler et al., 2005). Pathological anxiety is a core feature of Anxiety Disorders as defined in the Diagnostic and Statistical Manual of Mental Disorders–Fifth Edition (DSM-5), including Panic Disorder (PD), Generalized Anxiety Disorder (GAD), Social Anxiety Disorder (SAD), as well as of Posttraumatic Stress Disorder (PTSD). Patients with pathological anxiety experience symptoms of attentional dysfunction including hyper-vigilance, hyperarousal (Bogels and Mansell, 2004; Freeman et al., 2000; Joiner et al., 1999; Pflugshaupt et al., 2005; Richards et al., 2014) and neurocognitive disturbances of attentional bias to, and enhanced neural processing of, threat or negatively-valenced stimuli (Mogg et al., 2007; Ewbank et al., 2009).

There is considerable evidence indicating a link between increased locus coeruleus (LC) activity and the development of pathological anxiety in pre-clinical work. The LC are small, paired, longitudinally oriented, highly pigmented nuclei that are situated in the upper pons to each side of the fourth ventricle. The LC contain norepinephrine (NE)-elaborating neurons that project widely to cortical, subcortical and brainstem nuclei to rapidly and globally modulate arousal (Bremner et al., 1996a,b). The tonic LC-NE system sustains vigilance and orienting functions (David Johnson, 2003) and alerts or primes the organism to respond to any significant external event (Svensson, 1982). LC dysfunction has been associated with pathological cognitive states: lower than normal tonic activity is associated with hypo-arousal and attention deficits, whereas higher tonic firing is associated with hyper-arousal and anxious states (Howells et al., 2012). Indeed, in rodents, chronic or repeated stress increases LC-NE (Fan et al., 2014), adrenergic receptor expression (Li et al., 2018) and induces amplification of LC-NE reactivity to subsequent stressors (Jedema et al., 2001).

However, in human patients with pathological anxiety, evidence of LC-NE dysfunction has been indirect, partly due to limitations in discerning LC with 3-Tesla (3T) MRI. Early evidence indicated that patients with PTSD and PD given the alpha-2 receptor antagonist yohimbine, which increases LC-NE firing, show increased anxiety and panic symptoms (Southwick et al., 1993; Charney et al., 1984; Gorman et al., 1984), implicating a role for LC-NE in anxiety symptoms in humans. More recent studies in healthy volunteers indicate that anticipation of threat engages arousal and increases brainstem auditory evoked potentials (Baas et al., 2006) and pupil dilation (Clewett et al., 2018) in healthy volunteers, two responses thought to be indirect measures of LC activation. Two studies have shown LC activation using functional MRI in patients with PTSD (Naegeli et al., 2018; Morey et al., 2015), although these studies are limited by large voxel sizes and large smoothing kernels. Human studies implicating LC-NE dysfunction in other disorders of pathological anxiety are less numerous.

Previous studies of LC structural imaging in humans have utilized the T1-weighted turbo spin echo (TSE) technique, offering contrast in the LC due to the presence of neuromelanin (NM), a pigment structurally related to melanin that is synthesized from L-DOPA and found in large concentrations within LC neurons and other catecholaminergic cells. (Betts et al., 2019; Clewett et al., 2016). NM is MR-visible due to a magnetization transfer (MT) contrast mechanism. However, T1-TSE only indirectly captures MT contrast from off-resonance effects of alternate slice spin-echo imaging, a process that is time-consuming and limited by high energy deposition with high specific absorption rate (SAR), making it not possible with higher resolution MR. T1-TSE is also limited by large slice thickness (~3 mm), meaning that, while 3T MRI can be used to localize the LC in the upper pons (Sasaki et al., 2006), the characterization of LC size and morphology is suboptimal. Higher isotropic resolution is required to distinguish the signal from the LC from the signal of nearby nuclei. Ultra-high-field 7-Tesla (7T) MRI now provides the enhanced signal-to-noise ratio (SNR) and high spatial resolution required for differentiating the signal of LC from the signal of nearby pontine nuclei. In addition, high-field MRI can more directly measure NM by using MT MRI (Priovoulos et al., 2018), which provides vastly improved spatial resolution, acquisition time and SNR compared to 3T TSE. Having recently been approved by the Food and Drug Administration (FDA) for clinical use, 7T MRI is well tolerated and ideal for applications in imaging sub-milimeter brain structures not previously detectable *in vivo*.

This study aimed to use ultra-high field 7T MT MRI to localize the LC in humans with and without pathological anxiety, with 0.4 × 0.4 × 0.5 mm resolution in a feasible scan time. In addition, we aimed to apply a computational, data-driven LC localization and segmentation algorithm to delineate LC for all participants. The relationships between LC volume and trans-diagnostic measures of pathological anxiety and attentional control were subsequently examined in a dimensional approach based on the RDoC initiative, reflecting evidence that pathological anxiety is a trans-diagnostic construct (Wilamowska et al., 2010; Barlow et al., 2016; Nutt et al., 2002).

## 2 Methods and materials

### 2.1 Participants

All subjects were recruited through the Depression and Anxiety Center for Discovery and Treatment Disorders Program, Icahn School of Medicine at Mount Sinai. Patients were recruited across clinical disorders characterized by pathological anxiety including posttraumatic stress disorder (PTSD), panic disorder (PD), generalized anxiety disorder (GAD), social anxiety disorder (SAD), as well as healthy control (HC) subjects. Study eligibility was assessed with the following criteria: (1) male or female aged 18 to 55, (2) capacity to provide informed consent, (3) no history or current evidence of mental retardation or cognitive disorder, (4) no substance use disorder within the past year, (5) no active general medical problems, (6) no history of neurological disorder, (7) no current medications with known anxiolytic effects [including selective serotonin, serotonin-NE or NE reuptake inhibitors, benzodiazepines, pregabalin, or buspirone] at the time of the scan (8) no ferromagnetic metal present in the body and no other MRI risk factors. The following additional eligibility criteria are specific to study group: PTSD: (a) meets diagnostic criteria for current PTSD according to the DSM-5^92^, (b) Criteria A trauma of the civilian type (e.g., non-combat, threatened or actual interpersonal violence), (c) index Criteria A trauma occurred after the age of 18, (d) duration of current PTSD >12 months; PD: (a) meets diagnostic criteria for current PD according to the DSM-5, (b) duration of current PD >12 months; GAD: (a) meets diagnostic criteria for current GAD according to the DSM-5, (b) duration of current GAD >12 months; HC: no lifetime history of a psychiatric or neurological disorder or criteria A trauma. The study was approved by the Institutional Review Board at the Icahn School of Medicine at Mount Sinai. All subjects provided written informed consent before study entry and were compensated for their time.

### 2.2 Dimensional clinical measures

We employed dimensional clinical scales of anxiety and attentional control relevant across both patients and controls. All participants completed the Mood and Anxiety Symptoms Questionnaire (MASQ)^79^, a validated questionnaire that is based on the Tripartite model of Affect, proposed to account for comorbidity between depression and anxiety disorders (Watson et al., 1995); with 3 sub-scores: General Distress, Anhedonic Depression, and Anxious Arousal. All participants also completed the Adult Temperament Questionnaire (ATQ)^142^ that includes 3 sub-scores related to attentional control: attentional control, inhibitory control and activation control.

### 2.3 MRI acquisition

Participants were scanned using a 7T MRI scanner (Magnetom, Siemens, Erlangen, Germany) with a 32-channel head coil at the Leon and Norma Hess Center for Science and Medicine, ISMMS. Most subjects tolerated the MRI environment well. On entering the scanner, several subjects reported dizziness lasting 1–2 min, which they found tolerable.

**Fig. 1:**
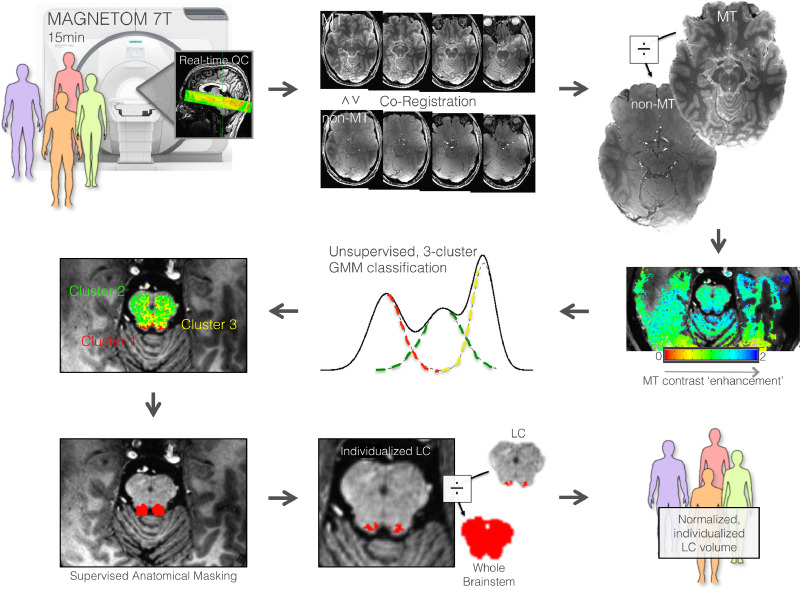
Schematic depiction of the workflow for computationally-derived locus coeruleus segmentation based on high-resolution MRI.

Structural T1-weighted dual-inversion magnetization prepared gradient echo (MP2RAGE) anatomical images were acquired first (repetition time (TR)=4500 ms, TE=3.37 ms, TI1=1000s and 3200 ms, flip angle (FA)=4 and 5°, iPAT acceleration factor=3, bandwidth (BW)=130 Hz/pixel, 0.7 mm isotropic resolution, whole brain coverage). Secondly, MT-MRI data were acquired with a 3-D segmented gradient-recalled echo (GRE) readout (turbo-FLASH; TFL) preceded by a train of 20 MT pulses of amplitude with 190 V transmit, and 7 min run time (Priovoulos et al., 2018). This approach at 7T gives 0.4 × 0.4 × 0.5 mm resolution with high SNR for upper pons and midbrain. The 3-D strategy enables a longer MT pre-saturation period versus 2-D and critically, MT-TFL is substantially more efficient in time and SAR than the current standard of T1-TSE (Uğurbil, 2014; Mougin et al., 2010). We used oblique slab imaging spanning the ventral tegmentum of the midbrain and pons. We additionally acquired an identical MT MRI resolution-matched [non-MT] TFL image for the purpose of NM signal enhancement computation (<5 min) (see Fig. 1). All images were visually inspected for signs of motion immediately after acquisition and repeated if necessary, to mitigate against deleterious effects on motion on this high-resolution protocol.

Human subjects were scanned with a Siemens Magnetom 7-Tesla MRI scanner for a ∼15min acquisition of magnetization transfer (MT) and non-MT scans spanning the midbrain. Real-time quality check (QC) was performed during acquisition for signs of motion. The scans were co-registered then the MT scan was divided by the non-MT scan creating a fractional map of specific MT contrast. Next, the MT contrast enhancement image was processed according to an unsupervised Gaussian mixture model (GMM) algorithm to classify each voxel into one of the three clusters based on three sources of MT contrast enhancement, including one cluster relating to neuromelanin (NM) content. The NM cluster was extracted with cylindrical masks lateral to the fourth ventricle, leading to voxel clusters attributed to the LC. Finally, the LC was normalized against whole brainstem volume for each subject, creating a normalized, individualized LC volume estimate for each individual.

### 2.4 LC localization and quantification

Here, we outline a computational method to delineating the LC that was fully automated, based on supervised and unsupervised machine learning techniques. This procedure harnesses the superior macromolecular signal separation, including NM, afforded by MT signal enhancement and utilizes fast computational signal distribution modeling.

Firstly, T1-weighted anatomical MRI data were processed using the FreeSurfer v.6.0 image analysis suite. MT-TFL images were co-registered to their T1-weighted anatomical image using a boundary-based technique (Saad et al., 2009). Next, a map of fractional MT enhancement was generated by dividing the MT by the non-MT TFL images, creating an image that is specifically enhanced for MT. This MT enhancement image was then computationally processed according to the Gaussian mixture modeling (GMM) clustering algorithm. The GMM computes a mixture of latent multi-dimensional Gaussian probability distributions for a given input dataset. Used in the classification mode, each voxel was assigned to one of the three classes based on the class to which it has the highest probability of belonging. A three-compartment model was used for sources of MT enhancement, for: fluid, NM, and white matter. Model parameters were estimated using an expectation–maximization algorithm consisting of two steps. First, the expectation (E) step computes the weights of the expected values of the latent variables in the model, assuming fixed model parameters. Second, the maximization (M) step updates the expected values for the previous parameter estimates that maximize the likelihood function, using the weights and based on all data points.

As predicted, mapping the middle compartment revealed NM-containing brainstem nuclei, including (dorsal to ventral in our imaging slab): substantia nigra, ventral tegmentum and LC,  in a data-driven manner, without imposing any user-defined thresholds or boundaries. To finally isolate the LC cluster itself, we took advantage of the anatomical definition of LC as lateral to the fourth ventricle, which is distinct in contrast from the other MT enhancing regions. Staying with the threshold-free approach, the fourth ventricle segment from FreeSurfer analysis of the coregistered T1 image was dilated 4 mm in all directions, and intersected with the compartment map of the LC enhancing regions, leading to the delineation of the LC volumes. The computationally-derived LC volume was visually inspected by an expert neuroanatomist (TN). We then quantified the MT-NM signal enhancement through a linear section of the brainstem in which the LC and adjacent pontine tegmentum are expected to be localized based on postmortem tissue (see Fig. 1), similar to previous studies (Priovoulos et al., 2018). Next, we quantified and compared the MT-NM signal enhancement in the region of the LC compared to non-LC, as defined by the computational GMM algorithm.

A group mean LC volume was computed from all subjects and compared with anatomically demonstrated LC dimensions determined by human postmortem histological delineations of LC (Sears et al., 2013), indicating a longitudinal extent of 14–15 mm (Fernandes et al., 2012; German et al., 1988) and a rostral origination at the inferior colliculus (German et al., 1988). The group mean LC volume was also visually compared to a standard space MRI delineation of LC (Keren et al., 2015).

### 2.5 Statistical group difference and correlational analyses

LC volume was computed as the sum of the number of voxels determined to be LC, multiplied by the image resolution (0.4 × 0.4 × 0.5). LC volume was then normalized against (divided by) the volume of the brainstem for each individual (LC_norm_) and entered into two-tailed independent samples t-test to compare between groups. LC volume was additionally normalized against whole-brain volume in confirmatory analyses. Secondly, LC_norm_ was correlated against the dimensional measures of anxiety (MASQ) and attentional control (ATQ) trans-diagnostically across the whole cohort using two-tailed Spearman correlation, controlling for age and sex separately. All tests were corrected for multiple comparisons using False Discovery Rate (FDR) correction.

## 3 Results

### 3.1 Participants

We applied ultra-high field quantitative MRI for the precise delineation of the LC *in vivo* in humans with and without pathological anxiety (Fig. 1). Twenty-nine subjects were scanned, including 14 non-psychiatric HC (10 male; mean age= 39.9 ± 9.0) and 15 patients with a DSM-5 anxiety or stress related disorders: 7 with PTSD (2 male, age=38.5 ± 3.5); 5 with GAD (3 male, age=38.6 ± 11.6); 2 with PD (females, age=26.0 ± 2.8); 1 with SAD (male, age = 49). Patients were clinically stable and not on any psychotropic medications at the time of scan. See Table 1 for subject characteristics. Subjects tolerated the 7T MRI scanner well. Two HC subjects (male, age 28–36) were excluded due to excessive motion (based on image blurring, combined with average motion for subsequent scan >1.2 mm). Motion for the remaining subjects (*N* = 27) was: average millimeter displacement = 0.002, 0.180, 0.043 mm (right, anterior, inferior), during a subsequent scan during which motion was recorded. Motion was not different between groups across 6 degree and displacement measurements (*p*>0.27).

**Table 1:** Demographic and clinical information.

Demographics	Healthy Control (*N* = 14)	Patients (*N* = 15)	p value
**Age, Mean (SD, range)**	39.9 (9.0, 28–57)	38.7 (10.2, 24–55)	0.756
**Gender, N Male (%)**	10 (71.4)	6 (40.0)	0.095
**Race/Ethnicity, N (%)**			
White/Caucasian	6 (42.8)	7 (46.6)	0.837
Black/African American	6 (42.8)	4 (26.7)	0.377
Hispanic/Latino	1 (7.1)	4 (26.7)	0.176
Undisclosed	1 (7.1)	0 (0.0)	0.309
**Self-report Scores, Mean (SD, range)**			
MASQ General Distress	11.4 (2.4, 10–18)	23.4 (10.0, 10–36)	<0.001
MASQ Anhedonic Depression	29.1 (8.9, 12–50)	37.8 (7.0, 21–49)	0.006
MASQ Anxious Arousal	12.6 (1.5, 10–17)	19.7 (10.3, 10–43)	0.018
ATQ-EC Activation Control	38.9 (6.2, 25–49)	31.9 (9.8, 16–49)	0.029
ATQ-EC Attentional Control	30.1 (6.3, 15–39)	18.0 (6.8, 6–29)	<0.001
ATQ-EC Inhibitory Control	38.3 (6.4, 25–48)	30.1 (7.7, 17–45)	0.004

### 3.2 High-resolution LC localization and quantification

The workflow for rapid, data-driven LC localization and segmentation is depicted in Fig. 1. The current approach at 7T gives 0.4 × 0.4 × 0.5 mm resolution with high SNR for upper pons and midbrain (see Supplementary Figure 1). A mixture of unsupervised algorithms and supervised anatomically-constrained processing was used to computationally delineate LC, utilizing the superior NM signal separation afforded by specific MT signal enhancement.

**Fig. 2:**
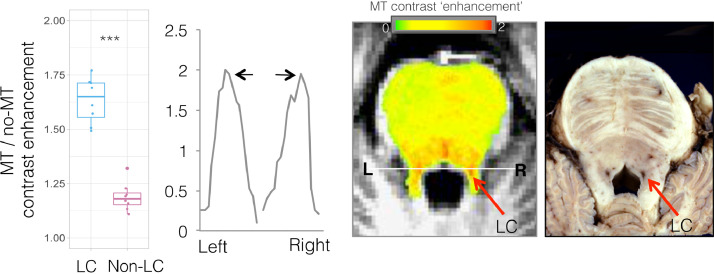
Magnetization transfer (MT) enhancement reveals neuromelanin-rich locus coeruleus (LC).

There was significant MT-NM signal enhancement in brainstem sections corresponding to the anatomic position of the LC observed in postmortem tissue, compared to adjacent pontine tegmentum (Fig. 2). The LC MT-NM signal enhancement was bilaterally symmetric (see Fig. 2 line plot), matching previous reports in a large sample of healthy volunteers (Shibata et al., 2006). The MT-NM signal enhancement separation for the LC compared to adjacent regions was less noisy than seen on previously demonstrated similar analyses that were based on MT-TFL acquisitions without MT-NM enhancement quantification (Priovoulos et al., 2018) (note the voxelwise signal enhancement intensity plotted from left to right across a line traversing LC and adjacent non-LC regions, Fig. 2). This reflects the specificity gain afforded by creating an MT-NM enhancement image by dividing the MT by the non-MT TFL images, compared to utilizing the MT-TFL image alone. This separation aids the computational delineation of LC, as noted previously (Priovoulos et al., 2018). We secondly quantified and compared the MT-NM signal enhancement in the region of the LC compared to non-LC, as defined by the computational GMM algorithm. There was significantly greater MT-NM signal enhancement in the region of the LC compared to non-LC regions as determined by the GMM LC quantification algorithm (*p* = 5 × 10^−9^, Fig. 2).

Left: Hyper-intensity of MT signal contrast ‘enhancement’ is plotted in the region of the LC compared to non-LC, as defined by computational GMM algorithm (left, ***p = 5 × 10^−9^). A line ROI was drawn through bilateral LC and the MT signal enhancement for each voxel along the line is plotted from left (L) to right (R), showing LC-related signal enhancement hyper-intensity (black arrows), facilitating data-driven LC delineation. Middle: Axial slices through the brainstem with overlaid red-yellow map of fractional MT enhancement due to neuromelanin content, calculated from MT/no-MT-turbo-flash MRI. Note hyper-intensity at the region of the LC (red arrows) in the magnified image, as well as other zones that could include dorsal raphe nuclei that have similar norepinephrine transporter levels as the LC. Right: Gross axial anatomic section through the formalin-fixed upper pons demonstrates the reddish cross-sections of the paired longitudinal columns of the LC to each side of the upper fourth ventricle. Histological image provided courtesy of T. Naidich.

**Fig. 3:**
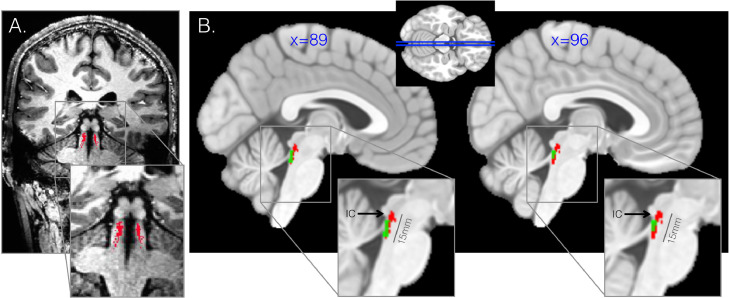
High-resolution locus coeruleus (LC) delineation.

An example high-resolution LC segmentation is demonstrated in Fig. 3A. The group mean LC volume spatially overlapped with a previously defined standard space MRI delineation of LC (Keren et al., 2015) (Fig. 3B). We found that the current approach corresponded well with previous histologically-defined anatomic definitions of the LC, which demonstrate an average LC length of 14–16 mm, a rostral boundary at the inferior colliculus (German et al., 1988), and an approximate bilateral volume of 112–120 mm (Fernandes et al., 2012; German et al., 1988) (Fig. 3B). Furthermore, we observed variation of LC volume in HC (mean=125.7 mm^3^, SD=59.3 mm^3^) that recapitulates prior findings of LC morphological variability (Fernandes et al., 2012; German et al., 1988; Keren et al., 2015) and coincides with histological evidence of human LC size as approximately 112–120 mm^3^ volume bilaterally (14–15 mm length, 2 × 2 mm width) (Fernandes et al., 2012; German et al., 1988). There was no hemispheric difference in LC volume across the sample (*p* = 0.98).

Ultra-high field 7-Tesla MRI provides 0.4 × 0.4 × 0.5 mm resolution enabling precise spatial localization of voxels that are assigned to the LC. A. Coronal slice through brainstem at the region of the LC for T1-weighted MP2RAGE MRI scan with the computationally-derived LC cluster overlaid for an example healthy subject. B. Group mean LC (red), showing similarity with expected length of LC 14–16 mm and expected rostral extent at the site of the inferior colliculus (IC). The current approach outperforms a standard template-space mask of the LC that was previously developed (green) by recapitulating the expected length and dorsal extent.

### 3.3 Normalized LC volume is higher in patients with pathological anxiety

**Fig. 4:**
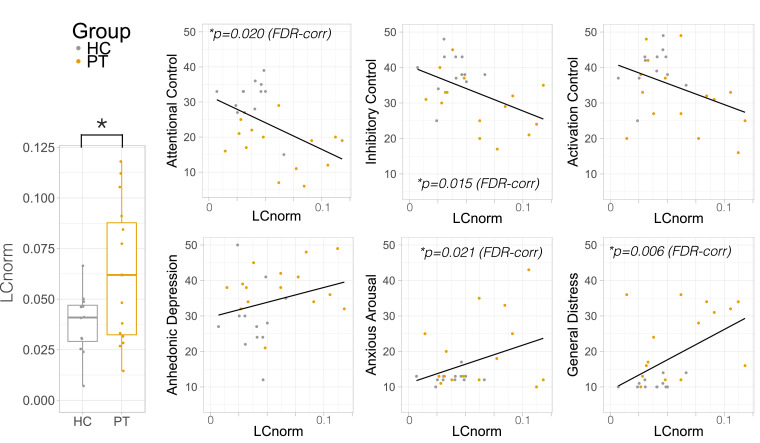
Locus coeruleus (LC) volume is higher in patients with pathological anxiety and is associated with shared symptoms.

LC_norm_ volume was larger in patients compared to controls (Cohen's *d* = 1.08, t_(20.5)_=2.44_,_ *p* = 0.024, Fig. 4). These findings remained significant when controlling for age (F_(1,24)_=4.99, *p* = 0.035) and trended towards significance when controlling for sex (F_(1,24)_=3.14, *p* = 0.089), an expected mediator of LC size (Bangasser et al., 2011; Valentino et al., 2012). Patients did not differ from each other based on categorical diagnosis (*p* = 0.712). LC volume normalized against whole-brain volume showed the same pattern, being larger in patients compared to controls (t_(20.5)_=2.30_,_ *p* = 0.032). There was no correlation between LC volume and whole-brain (*p* = 0.971) or brainstem volume (*p* = 0.288). MT-NM signal enhancement within the LC was not significantly different between groups, although there was a trend towards lower MT-NM signal enhancement in patients compared to controls (*p* = 0.065), which may relate to lower LC neurite integrity in patients.

Normalized LC volume (LCnorm) was higher in pathological anxiety (PT, gold) compared to healthy control subjects (HC, silver). LCnorm is plotted against self-reported attentional control (top, Adult Temperament Questionnaire – Effortful Control Questionnaire, ATQEC) and clinical symptoms (bottom, Mood and Anxiety Symptoms Questionnaire, MASQ) across all subjects trans-diagnostically. FDR= false discovery rate –corrected p-value.

### 3.4 Normalized LC volume is trans-diagnostically associated with lower attentional control and higher general distress

LC_norm_ volume was correlated with the dimensional measures of anxiety (MASQ) and attentional control (ATQ) trans-diagnostically across the whole cohort using two-tailed Spearman correlation, using FDR correction for multiple comparisons. LC_norm_ was negatively correlated with attentional control (*R*=−0.505, FDR-corrected *p* = 0.020), and inhibitory control (*R*=−0.545, FDR-corrected *p* = 0.015), across all subjects and controlling for age (Fig. 4). LC_norm_ was additionally positively correlated with general distress (MASQ) (*R* = 0.618, FDR-corrected *p* = 0.006) and anxious arousal (*R* = 0.483, FDR-corrected *p* = 0.021), across all subjects controlling for age (see Table 2 for statistics). There was no correlation between age and LC volume across all subjects (*R*=−0.17, *p* = 0.935). Controlling for sex did not impact these relationships, except the correlation with anxious arousal, which became a trend correlation (Supplementary Table 1).

**Table 2:** Two-tailed Spearman correlations between normalized locus coeruleus volume (LC_norm_) and self-reported clinically-relevant variables.

	ATQ-EC			MASQ		
	Activation Control	Attentional Control	Inhibitory Control	General Distress	Anhedonic Depression	Anxious Arousal
R	−0.357	−0.505	−0.545	0.618	0.349	0.483
p	0.079	0.01	0.005	0.001	0.088	0.014
FDR-corr p	0.088	*0.02	*0.015	*0.006	0.088	*0.021

## 4 Discussion

This was the first application of a high-resolution LC localization and segmentation protocol to delineate the LC in human subjects with and without pathological anxiety. Ultra-high field MRI was successfully utilized to maximize signal and contrast within the brainstem to reliably segment and measure the LC with 400 × 400 × 500 μm precision. Patients with pathological anxiety had larger LC volumes compared to healthy controls subjects and greater LC volume was dimensionally associated with higher anxiety and poorer attentional control across all subjects. This presents the first direct evidence linking LC volume to anxiety in humans *in vivo*.

The current computationally-derived LC was compared with a previously developed 3-Tesla MRI-based LC ROI (Keren et al., 2015) as well as postmortem histological staining studies of NE-containing and NM-containing LC neurons in the adult human brain (Fernandes et al., 2012; German et al., 1988). While the previously published MRI-based LC mask (Keren et al., 2015) extended approximately 7 mm in length, postmortem staining studies of LC neurons suggest longer LC spindles, ranging from 12–16 mm (Fernandes et al., 2012; German et al., 1988). Our computationally-derived LC extended approximately 14–15 mm on average for healthy controls, more closely matching these postmortem LC quantification studies (Fernandes et al., 2012; German et al., 1988). Furthermore, the current computationally-derived LC originated rostrally at the inferior colliculus, also coinciding with previous postmortem LC delineation studies (German et al., 1988). However, caution must be taken when comparing *in vivo* measurements and measurements from *ex vivo* formalin-fixed specimens, which might be expected to be lower than *in vivo* measurements.

While much preclinical evidence implicates the LC in the acute response to threat (Grant et al., 1984; Silveira et al., 1993; Hayley et al., 2001), and demonstrates its upregulation following chronic stress (Fan et al., 2014; Jedema et al., 2001; Sands et al., 2000; Keshavarzy et al., 2015), little work has directly demonstrated LC dysfunction in humans with pathological anxiety. This has been in part due to limited feasibility of discerning LC with 3T MRI. The current finding that larger LC size was associated with higher anxiety trans-diagnostically is consistent with recent work linking larger LC volume to greater negative memory recall (Hammerer et al., 2018). Patients with PTSD show higher LC functional MRI activation, exaggerated heart-rate responses, skin conductance and eye blink responses to loud sounds (Naegeli et al., 2018) as well as higher LC and insula functional MRI activation to fearful stimuli (Morey et al., 2015) compared to trauma-exposed controls. There is also evidence linking PTSD with increased LC connectivity with amygdala, striatum and insula during threatening eye gaze (Steuwe et al., 2015). This enhanced functional activation and connectivity may relate to larger volume but further studies linking structure and function are needed. In contradiction to the current findings, one post mortem study has indicated that the LC is smaller in veterans with probable PTSD (Bracha et al., 2005), although this was limited by a low samples size (*N* = 3) and forensic diagnoses of probable PTSD. Further studies are therefore required with larger sample sizes to explain these inconsistencies and quantify the LC volume across the spectrum of pathological anxiety disorders. Interestingly, female rats have larger and more complex LC than males (Bangasser et al., 2011; Valentino et al., 2012), with more sensitivity to corticotrophin releasing factor (CRF), which is linked with more hyperarousal and anxiety-like behaviors (Bangasser, 2013), providing evidence for links between LC size, integrity and anxiety-like symptoms.

There was a trend towards lower LC MT-NM signal enhancement in patients compared to controls, which may relate to reduced LC neurite integrity. Previous studies have shown that lower LC integrity, also measured by NM-related MRI signal, is associated with poorer memory for negative events (Hammerer et al., 2018) and lower verbal intelligence and cognitive reserve (Clewett et al., 2016) in healthy older adults. Together these findings suggest that larger LC volume and enhanced LC reactivity to aversive stimuli, as well as reduced neurite integrity within the LC could together contribute to symptoms underlying pathological anxiety, including higher anxious arousal, poorer memory and attentional control. Larger future studies are needed to tease apart the influence of gender and other vulnerability factors on LC volume and its relationship with anxiety.

LC volume was additionally associated with attentional control. This coincides with a wealth of pre-clinical evidence implicating LC in arousal and attention regulation (David Johnson, 2003; Howells et al., 2012; Aston-Jones et al., 1991). In the normal state and in the absence of threat, the tonic LC-NE system sustains vigilance and orienting functions (David Johnson, 2003), optimally around 1–3 Hertz tonic firing (Howells et al., 2012). The LC responds to all novel stimuli, mediates general attentional orienting (Usher et al., 1999) and becomes activated in states of heightened vigilance, when a disruptive stimulus requires reorienting behavior (Aston-Jones et al., 1991). Furthermore, the LC-NE system is thought to regulate associative learning in order to engender subsequent attentional biases (Ehlers and Todd, 2017). This process seems specific to threat: the LC-NE system mediates threat learning during an aversive event (Sears et al., 2013) and is involved in passive-avoidance memory consolidation (Chen et al., 1992) and aversive Pavlovian-to-instrumental transfer (Campese et al., 2017). Together this implicates a system whereby over-active LC responses to environmental cues or events trigger exaggerated fear-learning, attentional biases, hypervigilance and hyperarousal, all associated with the development or maintenance of pathological anxiety.

There are several limitations in the current study. While high-resolution MRI provides finer precision for assessing small neural structures, it is also limited by increased B1/B0 inhomogeneity and greater susceptibility artifacts, as well as higher sensitivity to motion. B1 inhomogeneity can be mitigated by using dielectric pads under the head, which cause redistribution of B1 and more signal uniformity. In addition, B1/B0 inhomogeneity and signal non-uniformity is usually restricted to lateral regions and air-tissue boundaries, with more uniform signal found around the brainstem and LC. Since these procedures are high-resolution, they are more impacted by subject motion. In the current study, 2 out of 29 (~7%) subjects were excluded due to motion, which acts as a relatively large amount of data loss. Head padding, subject instruction and short scan times can reduce motion, although physiologic motion may be more difficult to overcome and can be mitigated by cardiac or respiratory gating, which must be considered when developing sub-millimeter scanning protocols. Even given the limitation related to motion and increased susceptibility artifacts at higher field, recent work also demonstrates that high field 7T MRI benefits from improved SNR (Triantafyllou et al., 2005; Beisteiner et al., 2011; Morris et al., 2019), important for structural, functional and spectroscopy studies (Balchandani and Naidich, 2015).

This study demonstrated a high-resolution protocol for computationally defining the LC *in vivo* harnessing superior LC signal separation with 7T MRI based on specific MT signal enhancement and performed in a data-driven manner. We emphasize the trans-diagnostic relevance of LC volume across disorders of pathological anxiety and provide direct *in vivo* evidence linking LC structure to common psychopathology.

## CRediT authorship contribution statement

**Laurel S. Morris:** Conceptualization, Data curation, Formal analysis, Methodology, Writing - original draft, Writing - review & editing. **Aaron Tan:** Data curation, Formal analysis, Writing - review & editing. **Derek A. Smith:** Data curation, Project administration, Writing - review & editing. **Mora Grehl:** Data curation, Project administration, Writing - review & editing. **Kuang Han-Huang:** Data curation, Formal analysis, Writing - review & editing. **Thomas P. Naidich:** Data curation, Supervision, Writing - review & editing. **Dennis S. Charney:** Conceptualization, Funding acquisition, Supervision, Writing - review & editing. **Priti Balchandani:** Funding acquisition, Supervision, Writing - review & editing. **James W. Murrough:** Conceptualization, Funding acquisition, Supervision, Writing - review & editing. **Prantik Kundu:** Conceptualization, Funding acquisition, Methodology, Supervision, Writing - review & editing.

## Declaration of Competing Interests

In the past 5 years, Dr. Murrough has provided consultation services to Otsuka, Clexio Biosciences, FSV7, Boehringer Ingelheim, Sage Therapeutics, Novartis, Allergan, Fortress Biotech, Janssen Research and Development, Genentech, Medavante-Prophase, and Global Medical Education (GME) and has received research support from Avanir Pharmaceuticals, Inc. Dr. Murrough is named on a patent pending for neuropeptide Y as a treatment for mood and anxiety disorders. Dr. Charney is named as co-inventor on patents filed by the Icahn School of Medicine at Mount Sinai (ISMMS) relating to ketamine for the treatment for treatment-resistant depression, suicidal ideation and other disorders. ISMMS has entered into a licensing agreement with Janssen Pharmaceuticals, Inc. and it has and will receive payments from Janssen under the license agreement related to these patents for the treatment of treatment-resistant depression and suicidal ideation. PB is a named inventor on patents relating to magnetic resonance imaging (MRI) and RF pulse design. The patents have been licensed to GE Healthcare, Siemens AG, and Philips international.  PB receives royalty payments relating to these patents. The remaining authors disclose no conflicts of interest.

## Appendix Supplementary materials

Image, application 1

Image, application 2
