# Supplementary material for: Sub-millimeter variation in human locus coeruleus is associated with dimensional measures of psychopathology: An in vivo ultra-high field 7-Tesla MRI study
Source: Neuroimage Clin. 2020 Jan 15;25:102148. doi: 10.1016/j.nicl.2019.102148 (PMC7037543; doi:10.1016/j.nicl.2019.102148)
Supplement: Supplementary file 1 [file mmc1.docx]

Supplementary Table 1

|  | ATQ-EC |  |  | MASQ |  |  |
| --- | --- | --- | --- | --- | --- | --- |
|  | Activation Control | Attentional Control | Inhibitory Control | General Distress | Anhedonic Depression | Anxious Arousal |
| R | -0.182 | -0.41 | -0.411 | 0.499 | 0.238 | 0.353 |
| p | 0.385 | 0.042 | 0.041 | 0.011 | 0.253 | 0.083 |

***Supplementary Table 1. Two-tailed Spearman correlations between normalized locus coeruleus volume (LC_norm_) and self-reported clinically-relevant variables.*** *LC_norm_ was negatively correlated with attentional and inhibitory control subscales of the Adult Temperament Questionnaire (ATQ) and positively correlated with general distress as measured by the Mood and Anxiety Symptoms Questionnaire (MASQ), all controlling for sex.*
