# Supplementary material for: Sub-millimeter variation in human locus coeruleus is associated with dimensional measures of psychopathology: An in vivo ultra-high field 7-Tesla MRI study
Source: Neuroimage Clin. 2020 Jan 15;25:102148. doi: 10.1016/j.nicl.2019.102148 (PMC7037543; doi:10.1016/j.nicl.2019.102148)
Supplement: Supplementary file 2 [file mmc2.docx]

Supplementary Figure 1 Legend.

Participants were scanned using a 7T MRI scanner. Magnetization transfer (MT) MRI data were acquired with a 3-D segmented gradient-recalled echo readout preceded by a train of 20 MT pulses of amplitude with 190V transmit. This approach at 7T gives 0.4x0.4x0.5mm resolution with high SNR for upper pons and midbrain.
